# Imaging of Ganglioneuroma: A Literature Review and a Rare Case of Cystic Presentation in an Adolescent Girl

**DOI:** 10.3390/diagnostics13132190

**Published:** 2023-06-27

**Authors:** Giulia Pacella, Maria Chiara Brunese, Federico Donnarumma, Michele Barrassi, Fabio Bellifemine, Guido Sciaudone, Gianfranco Vallone, Germano Guerra, Giuseppina Sallustio

**Affiliations:** 1 Department of Medicine and Health Sciences “Vincenzo Tiberio”, University of Molise, 86100 Campobasso, Italy; mariachiarabrunese@gmail.com (M.C.B.); f.bellifemine@studenti.unimol.it (F.B.); guido.sciaudone@unimol.it (G.S.); gianfranco.vallone@unimol.it (G.V.); germano.guerra@unimol.it (G.G.); 2 Fondazione Potito, 86100 Campobasso, Italy; federico.donnarumma@hotmail.it (F.D.);; 3 Department of Radiology, Cardarelli Hospital, 86100 Campobasso, Italy; michelebarrassi@yahoo.it

**Keywords:** retroperitoneal mass, cystic mass, ganglioneuroma, CT, MRI, backpain

## Abstract

Retroperitoneal ganglioneuroma is a rare neuroectodermal tumor with a benign nature. We performed a literature review among 338 studies. We included 9 studies, whose patients underwent CT and/or MRI to characterize a retroperitoneal mass, which was confirmed to be a ganglioneuroma by histologic exam. The most common features of ganglioneuroma are considered to be a solid nature, oval/lobulated shape, and regular margins. The ganglioneuroma shows a progressive late enhancement on CT. On MRI it appears as a hypointense mass in T1W images and with a heterogeneous high-intensity in T2W. The MRI-“whorled sign” is described in the reviewed studies in about 80% of patients. The MRI characterization of a primitive retroperitoneal cystic mass should not exclude a cystic evolution from solid masses, and in the case of paravertebral location, the differential diagnosis algorithm should include the hypothesis of ganglioneuroma. In our case, the MRI features could have oriented towards a neurogenic nature, however, the predominantly cystic-fluid aspect and the considerable longitudinal non-invasive extension between retroperitoneal structures, misled us to a lymphatic malformation. In the literature, it is reported that the cystic presentation can be due to a degeneration of a well-known solid form while maintaining a benign character: the distinguishing malignity character is the revelation of immature cells on histological examination.

## 1. Introduction

Ganglioneuroma (GN) represents 0.7–1.6% of primary retroperitoneal solid masses. It is a benign neuroectodermal neoplasm formed by ganglia and Schwann cells into a variable amount of myxoid stroma, ganglion cells, and nerve fibers; immature neuroblastic elements are not included. [1,2]. Ganglioneuroma is found along the distribution sites of the sympathetic nervous system, particularly in the posterior mediastinum and retroperitoneum, in young adults, with a predilection for the female sex, and in the adrenal medulla in the age groups 2–4 years and 40–50 years [3,4]. Although GN is considered a benign tumor, the mass size can increase, causing compressive symptoms on the adjacent structures and tissues, as major vessels or nerves [5]. For this typical behavior, the most common clinical presentation is represented by pain, often, in the case of retroperitoneal mass, back pain [6]. For these patients, surgery is the most appropriate treatment strategy, and in most patients, an R0/R1 resection is curative. A few patients have reported recurrence in a follow-up of three years, but it is extremely rare [6,7]. In some cases, in which mass cells are not completely differentiated at all, the risk of malignant transformation is higher, therefore an accurate radiological differential diagnosis is mandatory [8,9]. However, in a retrospective observational study, the malignant transformation from ganglioneuroma to gangliobastoma was described as rarer than recurrence, involving less the 1% of patients included. Several studies and case reports have already described the most frequent radiological features of ganglioneuroma, but the very well-known “whorled” appearance of ganglioneuroma on MRI T2 images, considered almost pathognomonic, was not so frequently described in clinical practice and reported in observational study only in the 30% of cases. Therefore, the radiological diagnosis of ganglioneuroma is still a key point and appears challenging for radiologists and clinicians due to many atypical cases [10]. Furthermore, mixed and immature forms are described in the literature, where ganglioneuroma was frequently associated with pheochromocytoma, and different grades of cell maturations are reported [11,12].

Due to the existence of different histologic forms and the possibility of developing ganglioneuroma in different anatomic sites, the imaging pattern can be variable and many atypical forms exist [13,14,15].

Cystic degeneration is more frequent in schwannomas, even if it cannot be considered a characteristic feature [16]. This uncommon pattern is due to a significantly increased lesion volume including cyst formation, central necrosis, calcification, and hemorrhage [17].

According to the International Neuroblastoma Pathology Classification, Ganglioneuroma-Maturing is composed predominantly of a Schwannian stroma and mature ganglion cells [18].

Furthermore, few studies have specifically focused on the differential diagnosis of retroperitoneal masses, which is necessary to plan the most appropriate diagnostic and treatment strategy. To date, CT and MRI, also due to innovation in 3D reconstructions, can give a high suspicion of malignant behavior and can identify ganglioneuromas through their typical features [6]. CT and MRI can also identify the complication of compressive syndromes related to masses usually wider than 10 cm [6]. This review and case report aims to describe the radiological features of ganglioneuroma on CT and MRI images, comparing them in typical and even atypical presentations.

## 2. Case Presentation

**Figure 1:**
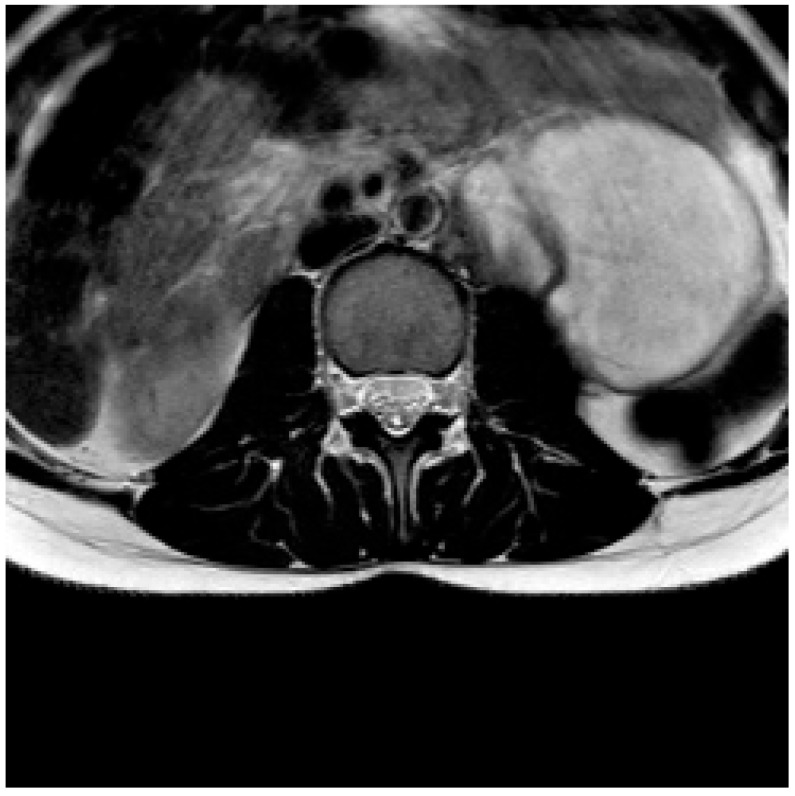
The axial T2-weighted sequence shows a cystic retroperitoneal paravertebral left mass as an occasional finding.

We report a case of an 18-year-old female patient, presenting only the symptom of significant recent back pain, who underwent a lumbosacral spine MRI. The survey and the axial T2-weighted MRI sequences show a multiloculated cystic mass, with a maximum axial diameter of about 7.5 cm and longitudinal extension of about 10 cm, in the left retroperitoneal site, with predominantly sub-renal development and a wide contact surface with the ipsilateral psoas muscle, hyperintense in the T2-weighted sequences then predominantly fluid. We also report a pelvic effusion of about 3.5 cm thickness (Figure 1).

The exam is inappropriate for a relevant characterization of the lesion; therefore, a dedicated complete abdominal MRI was performed using TSE T2, DUAL, STIR, DWI, and THRIVE sequences, with multiplanar imaging acquisition, before and after administration of paramagnetic contrast agent.

**Figure 2:**
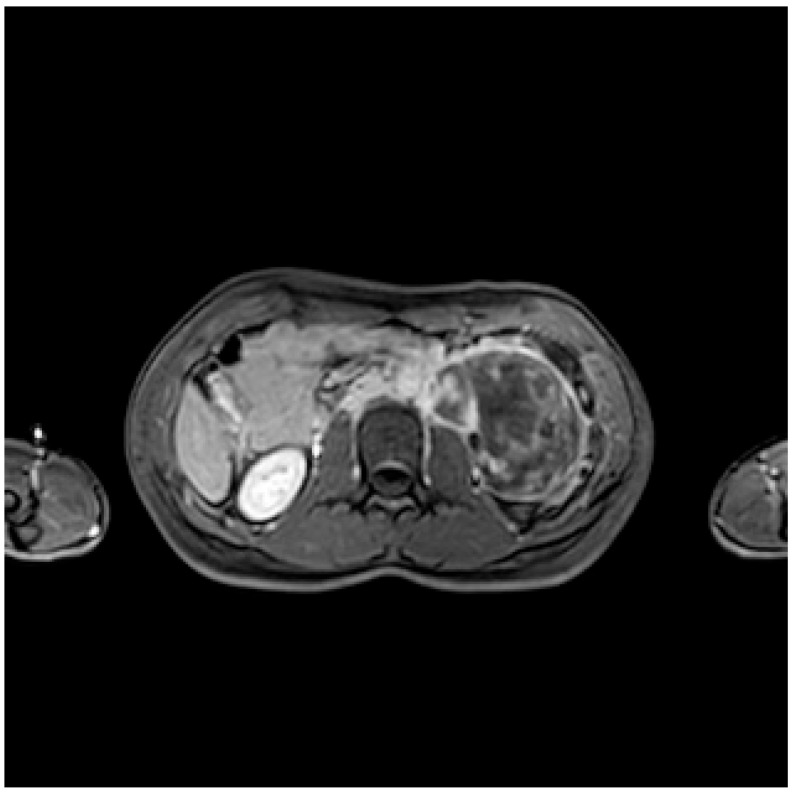
The T2-weighted fat-saturated sequence shows a hyperintense signal of the lesion with a medial hypointense dishomogeneity.

The exam confirms the presence of a retroperitoneal fusiform expansive formation in the left anterior pararenal space, approximately 11 × 8 × 7.4 cm; the major longitudinal axis extended from an axial plane passing through the renal hilum and the pancreatic tail. It confirms the wide contact surface with the ipsilateral psoas muscle. The lesion displaces the kidney posteriorly, but it spares the adjacent parenchyma and vascular structures. The MRI also confirmed the multilocular cystic appearance, hypointense in T1-weighted sequences and hyperintense in the T2-weighted ones, and added a more heterogeneous pattern in the medial component (Figure 2).

**Figure 3:**
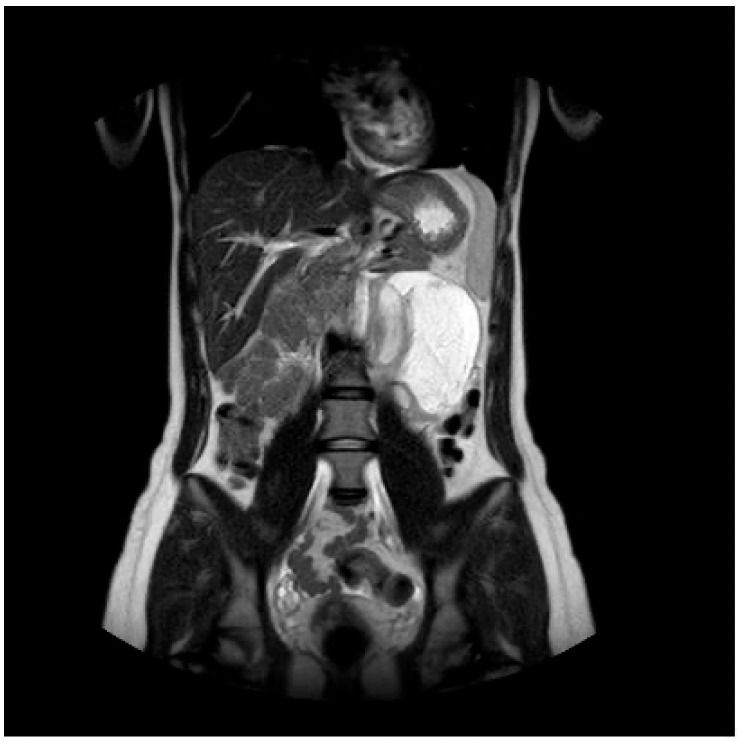
Ganglioneuroma in a coronal-T2 image.

**Figure 4:**
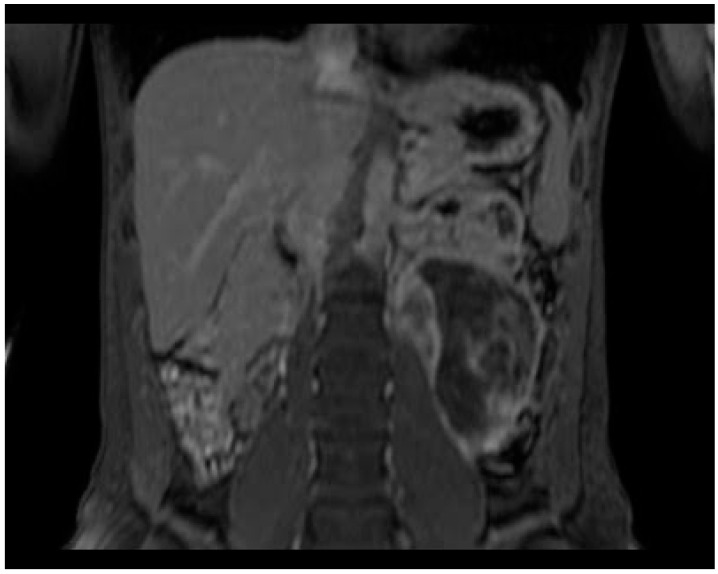
On coronal T1-spir image the ganglioneuroma shows a delayed phase enhancement.

**Figure 5:**
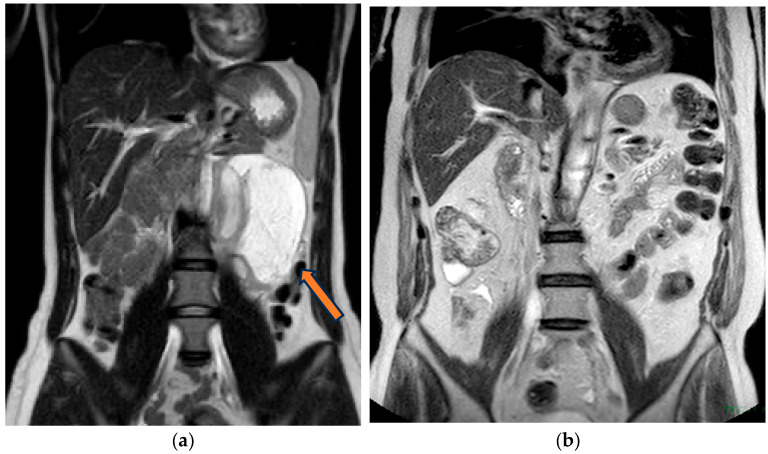
(**a**) patient with cystic ganglioneuroma (arrow); (**b**) a different patient without any retroperitoneal mass.

The dynamic contrast study shows late contrast enhancement of the intra-cistic septa and of the wall. In the STIR sequences, there is no suppression of the central component signal, and in the DWI/ADC weighted sequences, there are no evident signs of restricted proton diffusivity (Figure 3, Figure 4 and Figure 5).

The MRI characteristics point toward the suspicion of a disontogenic cyst or cystic lymphangioma [2].

After laparoscopic surgical exploration, the para-aortic paravertebral retroperitoneal mass has the appearance of a white neoformation with defined margins, and a good cleavage plane that allows complete excision.

The histological characterization establishes the diagnosis of ganglioneuroma with maturative aspects, a lesion microscopically constituted by fascicles of spindle cells of Schwannian aspect (S100+ and SOX10+), mixed with scattered ganglion elements (synaptophysin+), also arranged in small clusters, of different degrees of maturation but without significant proliferative and mitotic activity. There are aspects of myxoid degeneration and occasional calcifications and chronic inflammation.

The first imaging follow-up was carried out 3 months later. An abdominal MRI exam was performed with the same pre-operative study technique. In the surgical site, no MRI signs of residual disease or abdominal–pelvic lymphadenopathy are documented; only a residual pelvic effusion of 2 cm thickness is reported.

## 3. Literature Review

We searched the PubMed database for the following keywords: retroperitoneal mass; ganglioneuroma; MRI; CT; diagnosis. We considered the papers published until April 2023. Articles were first filtered and chosen by title and abstract, then a full-text evaluation was performed. Case series with a minimum of four patients were included. Reviews, letters, and case reports were excluded. We included only articles in the English language. Matching our keywords, we found a total of 338 papers. We excluded 329 papers because they did not treat the imaging features of retroperitoneal ganglioneuroma, or they were labeled as reviews or case reports. Three articles were excluded because they were not in the English language. We included nine studies. The included studies performed a pre-operative diagnosis through CT or MRI. The radiological features considered were tumor size (axial and coronal or longitudinal diameter), shape, calcification, and enhancement [6,12,13,17,19,20,21,22,23]. The tumor size reported in the studies was defined by the three diameters (axial × anteroposterior × longitudinal) or the largest diameter; among the measurements reported, ganglioneuroma was in the range of 3–24 cm, but most of the ganglioneuromas were smaller than 10 cm [6,10,11,12,13,14,15,16,17]. Ganglioneuroma appears as a solid paravertebral mass with a longitudinal orientation, often well-circumscribed [6,12,13,17,19,20,21,22,23]. In the study by Qian-Wen Zhang et al., of 43 lesions, 23 were oval, 15 were lobulated, and only 5 were irregular on both CT and MRI [5]. Calcifications are common on CT images and the discrete and punctate pattern of distribution is useful for the differential diagnosis of ganglioneuroblastoma and neuroblastoma, even if in the study by Ichikawa et al. a ganglioneuroma with a coarse pattern of calcification has been described [6,15,19]. On CT images, the enhancing pattern progressively grows in the delayed phase, but a small percentage with no enhancement has also been reported [6,12,13,17,19,20,21,22,23]. On MRI images, the components show a homogeneous signal of intensity lower than the liver on T1-weighted images, while the intensity signal is higher and heterogeneous on T2-weighted images [6,12,13,17,19,20,21,22,23]. Y. Zhang et al. found two cases of low–intermediate signal on T1 weighted images, in ganglioneuromas with a fat component or hemorrhage [19]. Cystic or irregular mixed density is considered atypical in several studies, but it can be due to a cystic degeneration more typical of Schwannomas [6,12,13,17,19,20,21,22,23]. However, the transition from benign to malignant is possible and reported in few cases, thus, the different grade of maturation, and maturative ganglioneuroma is the most differentiate form [6,12,13,17,19,20,21,22,23]. MRI enhancement in post-contrast T1-weighted phases appears more heterogeneous in patients with a large amount of myxoid stroma [21]. In the study by Qian-Wen Zhan et al., this variability was reported in 6/19 patients who show homogeneous enhancement [6]. In dynamic MR studies, the enhancement gradually increases, except for in the case of degenerative tumor with an early enhanced pattern, followed by a partial wash-out of contrast material [6]. Among the MRI features, it is known that the “whorled sign” is considered typical of ganglioneuroma. This sign is shown by the tumor as a high intensity in the T2-weighted image with a part showing low-signal and nodular aspects. The whorled sign corresponds to the tumor’s Schwann cells and collagen fibers [6]. The myxoid matrix shows late enhancement in the extracellular space; adipocytic components or cystic evolution are rarely reported [23,24,25,26]. In the study by Qian-Wen Zhang et al., the whorled sign was present in 14/19 cases [6]. The study conducted by Qian-Wen Zhang et al. proposed the “vessel encasement sign”, evident when the lesion envelops major vessels, classified into three types: tumor surrounding the coeliac axis or the superior mesenteric; tumor surrounding at least one of renal pedicles; tumor surrounding the aorta or vein cava [6].

## 4. Discussion

The appearance of ganglioneuroma in imaging can be very variable because it depends on the composition of the lesion, in particular the presence of myxoid tissue, collagen fibers, and varied cells [6,12,13,17,19,20,21,22,23,24,25]. The features are more variable in MRI images than in CT, where calcifications are related to the benign nature of the lesions [6,12,13,17,19,20,21,22,23,24,25]. The complications caused by the growth of the mass are rare and are represented by mass-effect, and this may even imitate deep thrombosis for compression [6,12,13,17,19,20,21,22,23,24,25]. It is also accurately detected with CE-CT to start the appropriate treatment [27,28].

In our case, the paravertebral site of the lesion and the heterogeneous aspect in T2-weighted sequences could have oriented towards a neurogenic nature, however, the predominantly cystic or fluid aspect and the considerable longitudinal non-invasive extension between retroperitoneal structures mislead to a lymphatic malformation. In the literature, it is reported that a ganglioneuroma could show cystic presentation due to the degeneration of a well-known solid form while maintaining a benign character: the distinguishing malignity character is the revelation of immature cells on histological examination [29].

The differential diagnosis is still changing and includes neuroblastoma and ganglioneuroblastoma from the same cell line, ganglion cells, but also schwannoma, lymphangioma, lymphoma, or dysontogenic cysts [6,12,13,17,19,20,21,22,23,24,25].

The malignant tumors, ganglioneuroblastoma, and gangliobastoma, often develop in children under 2 years old [9,21]. They have also an irregular shape, without a homogeneous pattern, and may present necrosis and hemorrhage. They also present a coarse pattern of calcifications rather than the discrete punctate pattern of ganglioneuroma [6,12,13,17,19,20,21,22,23,24,25].

Schwannoma is the most common retroperitoneal tumor. It originates from the nerve sheath and it often has a round shape rather than the oval or elongated shape of ganglioneuroma. It may develop cystic degeneration or calcifications. It can show a different behavior from ganglioneuroma in T2-weighted images, with a central zone of low intensity [23,25,26].

Lymphangioma is more common in males and rarely presents enhancement, and it often appears as unilocular or multilocular thin-walled cysts [23,25,26].

Retroperitoneal mass MRI characterization requires a differential diagnosis algorithm that first excludes the most frequent histotypes, such as lymphomas, representing about 1/3 of retroperitoneal lesions, and more expansive and infiltrating sarcomas. The most frequent solid type of lesions should also be excluded: well-differentiated liposarcoma, whose distinctive MRI feature is the adipocytic component, and leiomyosarcoma, whose distinctive feature is the vascular invasiveness [29].

On T2-weighted sequences, a lesion showing peripherical hyperintensity and central hypointensity is suspicious for a neurogenic origin, especially in paravertebral localization. Moreover, vascular enhancement is increased in the paraganglioma and reduced in the presence of a myxoid matrix.

Finally, cystic lesions, especially if they are longitudinally extended and without invasion of nearby structures, are reliable in the first hypothesis of lymphatic malformations [23,30].

However, the absence of neuroblasts on histological examination is the only certain exclusion element for a malignant histotype, such as ganglioneuroblastoma or neuroblastoma [25,31].

Surgical resection of benign histotypes such as ganglioneuroma is the only effective therapy, even if it may be complicated by the involvement of surrounding structures [32,33].

The Transatlantic Australasian Retroperitoneal Sarcoma Working Group conducted a retrospective study on 328 patients affected by abdominopelvic and retroperitoneal ganglioneuroma. Most of the patients (64.6%) underwent surgery, while 35.4 underwent active surveillance. In the follow-up, the malignant transformation was rare, but three cases out of 328 patients (0.9%) evolved into neuroblastoma. However, the patients who underwent active surveillance had stable disease in more than 90% of cases. In a 3-years follow-up, 84.4% of patients who had undergone surgery were disease-free. Recurrence was considered as rare as malignant transformation, occurring in only four patients (1.9%) [33].

In the last 20 years, minimally invasive surgery has been affirmed in general surgery, almost becoming the standard of care for benign but also for oncological diseases [7,34,35].

In this field, several studies have affirmed that minimally invasive surgery provides a shorter length of stay and a decrease in complications [36,37,38]. In the case of undiagnosed forms or coexistence of two benign conditions, successful outcomes are reported also for advanced oncological disease and simultaneous resections [39,40,41].

Currently, surgical procedures for retroperitoneal mass, and adrenal tumors above all, are safe and diffuse, and new technologies have been developed [42,43,44,45].

Artificial intelligence has already been tested on retroperitoneal masses to improve diagnosis, prognosis, and management of treatment strategies [46,47,48,49]. Additionally, for elderly patients with good performance status, surgery can be the best treatment strategy [50,51].

Concerning differential diagnosis, machine learning has been tested in a few studies to differentiate neuroblastoma from other tumors with a lower malignancy, like ganglioneuroma and ganglioneuroblastoma [52,53,54].

Due to the complexity of the anatomy of the retroperitoneum, AI also aims to help clinicians to perform CT-guided biopsies. This technique aims to avoid any damage to vascular structures [55,56].

As for other tumors, AI will be fundamental in recognizing the number of undifferentiated cells can be a key point in diagnosis and treatment and even the improvement of the standard technologies available [57].

Further AI studies will focus on achieving an accurate diagnosis through non-invasive exam, avoiding sedation for children during MRI exams, as ultrasound, or with short MRI protocol improved by artificial intelligence software.

## 5. Conclusions

This case reminds us that MRI characterization of a primitive retroperitoneal cystic mass should not exclude the occurrence of an atypical cystic presentation of benign tumors as ganglioneuroma or schwannoma; in the case of ganglioneuroma, the first suspicion sign should be the paravertebral localization.

## Data Availability

Data available on request due to restrictions, e.g., privacy or ethical. The data presented in this study are available on request from the corresponding author. The data are not publicly available due to the presence of other sensitive information not included in the study.
